# Oral Glutamine Supplement Inhibits Ascites Formation in Peritoneal Carcinomatosis Mouse Model

**DOI:** 10.1155/2013/814054

**Published:** 2013-09-30

**Authors:** Ming-Jen Chen, Tsang-En Wang, Shu-Jung Tsai, Ching-Chung Lin, Chia-Yuan Liu, Horng-Yuan Wang, Shou-Chuan Shih, Yu-Jen Chen

**Affiliations:** ^1^Division of Gastroenterology, Department of Internal Medicine, Mackay Memorial Hospital, Taiwan; ^2^Mackay Junior College of Medicine, Nursing and Management, Taipei, Taiwan; ^3^Mackay Medical College, New Taipei, Taiwan; ^4^Department of Radiation Oncology, Mackay Memorial Hospital, Taipei, Taiwan; ^5^Graduate Institute of Pharmacology, Taipei Medical University, Taipei, Taiwan

## Abstract

*Background*. Peritoneal carcinomatosis (PC) accompanied with ascites formation causes several distressing symptoms, resulting in poor quality of life. *Methods*. Twenty BALB/c nude mice generated by direct orthotopic injection of human pancreatic cancer PANC-1 cells were randomized to receive either a stock laboratory diet or a stock diet supplemented with glutamine. Half of the mice were sacrificed at day 76 to measure the amount of ascitic fluid and pancreatic tumor volume. The remaining mice were subject to survival analysis. Serum albumin levels were estimated every 2 weeks. *Results*. At day 76, the average amount of ascitic fluid measured in the control group was 1.2 ± 0.3 mL compared to 0.5 ± 0.5 mL from the glutamine-supplemented mice (*P* = 0.045). The volume of pancreatic tumor was 2.60 ± 0.8 cm^3^ in the control group and 1.98 ± 1.3 cm^3^ in glutamine-supplemented mice (*P* = 0.39). The mean survival time of glutamine-supplemented mice was prolonged from 87 ± 4 to 101 ± 2 days (*P* = 0.0024). Mean serum albumin levels were higher in the glutamine-supplemented group. *Conclusions*. This preclinical study showed that oral supplementation of glutamine may provide ascites-reducing activity in pancreatic cancer patients with PC, via a cell-mediated immunity-independent mechanism.

## 1. Introduction

Peritoneal carcinomatosis (PC) is well established as a terminal feature of advanced primary or secondary neoplasms involving the peritoneum. PC is a challenging complication associated with a poor prognosis and limited treatment options [1]. Locally advanced pancreatic cancer is one of the most common diseases causing PC and subsequent ascites formation. Terminal stage cancer patients with PC have an estimated median survival of 3–6 months [2]. At this terminal stage, quality of life, rather than the prolonging of survival, is considered the most crucial issue in palliative care. Nevertheless, the importance of life-prolonging palliative care is gaining recognition in the new era of cancer management.

Because PC-associated ascites develops due to hydrostatic pressure factors rather than osmotic factors, the current management of the condition includes abdominal paracentesis, diuretics administration, and salt restriction. Nutrition support represents an alternative strategy to improve the general well-being after management for ascites. However, the value of nutrition support in patients with PC remains controversial with inconclusive survival benefits [3] and concerns that it may accelerate tumor growth [4, 5].

The pathogenesis of ascites formation in PC is unclear. It is thought to be a correlation between endothelial cells, angiogenesis, and cytokines released in the peritoneum [6–8]. Endothelial-leukocyte interactions certainly play a role resulting in the release of cytokines and in activation of the endothelium of the peritoneum. A previous experimental study suggested that injection of genetically modified fibroblasts expressing interleukin-12 is an effect treatment for murine pancreatic PC via activated innate immunity, and it particularly activated M1 macrophages [9]. Another study found that angiopoietin-1, an endothelial cell regulator, could reduce ascites formation by mediating vascular endothelial growth factor [10].

Glutamine (GLN), the most abundant free amino acid in circulation, serves as an important energy source for many rapidly proliferating cells, especially enterocytes, lymphocytes, and fibroblasts. Various randomized controlled trials in noncancer patients have shown that GLN improved nitrogen metabolism and maintained the immune response, thus improving patient outcome [11, 12]. 

The goal of the present study was to evaluate the potential effects of orally administered GLN in tumor-bearing hosts with PC and ascites.

## 2. Methods

### 2.1. Animal and Experimental PC Model

**Figure 1:**
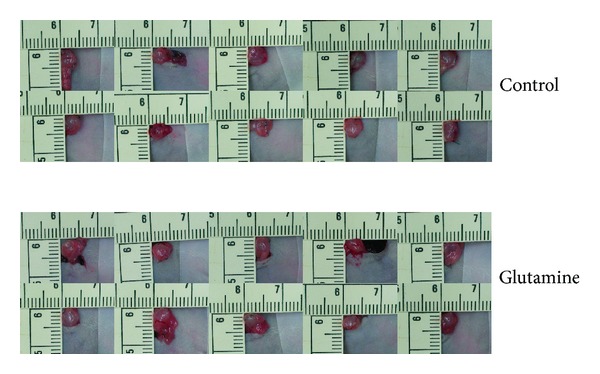
Experimental PC was generated by direct orthotopic injection of PANC-1 cells into the pancreatic tail. Animals underwent laparotomy to confirm tumor growth reaching up to 5 mm on the 10th day.

Male BALB/c nude mice, between 6 and 8 weeks old, were used in accordance with institutional guidelines. PANC-1 cells, derived from a poorly differentiated human pancreatic epithelioid carcinoma, were purchased from the American Type Culture Collection (ATCC, Rockville, MD, USA). PANC-1 cells were harvested at a concentration of 5 × 10^6^/mL from subconfluent cultures. Experimental PC was generated by direct orthotopic injection of PANC-1 cells into the pancreatic tail. To prevent leakage, a cotton swab was gently held for 1 min over the site of injection. The abdominal wound was then closed with sutures. Animals received laparotomy to confirm tumor growth reaching up to 5 mm on the 10th day (Figure 1).

### 2.2. Treatment Protocols

Twenty mice with confirmed tumor growth at day 10 were randomized into 2 groups with a similar average body weight in each. Group A (*n* = 10) received a stock laboratory diet with vehicle for glutamine, and Group B (*n* = 10) received a stock laboratory diet supplemented with 100% GLN (5 mg/(kg*·*day)). 

### 2.3. Tumor Growth, Serum Albumin, Aspartate Aminotransferase Level, and White Blood Counts, Ascites Formation, and Survival

Five mice from each group were sacrificed at day 76. Ascites were collected by performing a lower midline incision, completely draining the intraabdominal fluid, and the amount of ascetic fluid was measured using a 0.5 mL insulin syringe. Incisions were then extended to allow observation of the peritoneal cavity and confirmation of the extent of PC. Tumors were excised, and the tumor volume was measured as 1/2 × *a* × *b* ^2^ (*a* = the maximal diameter and *b* = the minimal diameter). The remaining 5 mice in each group were then observed until death for survival analysis. Serum albumin, aspartate aminotransferase levels (AST), and circulating white blood counts (WBC) for each animal were collected every 2 weeks. This study received approval from the Institutional Animal Care and Used Committee and the Institutional Review Board of Mackay Memorial Hospital, Taipei, Taiwan. 

## 3. Results

**Figure 2:**
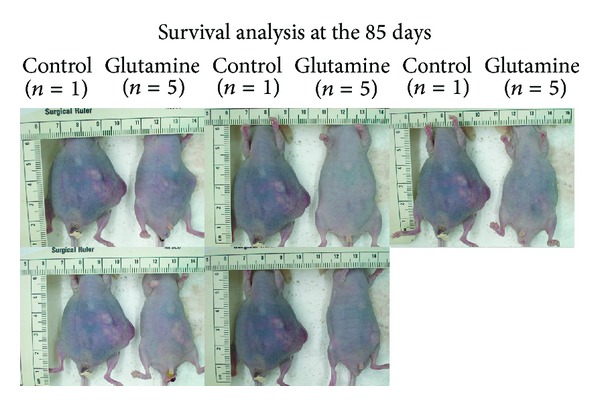
The remaining 5 mice in each group were observed until death for survival analysis. At day 85, only 1 animal in the control group was alive compared to all 5 mice in the GLN-treated group. The abdominal circumference was obviously less in the GLN-treated mice.

**Figure 3:**
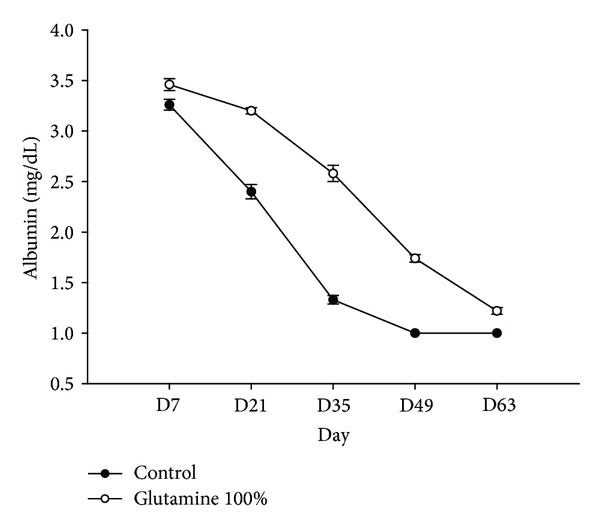
The mean serum albumin level was higher in the GLN-treated group.

All mice tolerated the treatment well. The amount of ascites collected was 1.2 ± 0.3 and 0.5 ± 0.5 mL in the control and GLN-treated group, respectively, (*P* = 0.045). At day 76, the volume of the pancreatic tumor was 2.60 ± 0.8 cm^3^ in the control mice and 1.98 ± 1.3 cm^3^in the GLN-treated group (*P* = 0.39). The remaining 5 mice in each group were then observed until death for survival analysis. At day 85, only 1 animal from the control group was alive compared to all 5 mice in the GLN-treated group. The abdominal circumference was obviously less in the GLN-treated mice (Figure 2). The mean survival period of the mice (*n* = 5) was longer at 101 ± 2 days with GLN supplementation in contrast to 87 ± 4 days in the control group (*P* = 0.0024). The mean serum albumin level was higher in GLN-treated group for each blood sample collected (Figure 3). There were no significant difference of circulating WBC and serum AST level on both groups. 

## 4. Discussion

Immunonutrients are specific nutrients that exert immunological effects when consumed at levels above the daily requirement. GLN is a well-known immunonutrient because it is essential for the growth and function of T lymphocytes and natural killer cells, both of which have been shown to suppress cancer progression through their cytolytic activity [13]. Orally administered supplementation of GLN was shown to prevent breast tumor growth by inhibiting production of the antioxidant glutathione, activating p53 signaling, and inhibiting PI3 K/Akt signaling [14, 15]. In the athymic mice model used in our study, we demonstrated a beneficial role of GLN in reducing ascites formation, slowing tumor growth, and prolonging survival. There was no significant difference on circulating WBC in these two groups. Given that T-cell-mediated immunity is insufficient in athymic mice, the beneficial effects of GLN supplementation are likely to result via a mechanism independent of T-cell-mediated immunity. However, the impact of GLN on immune cells infiltrating in ascites remains to be examined in immune competent experimental animals.

In this preliminary study, orally administered supplementation of GLN reduced the amount of ascites produced in PANC-1 tumor-bearing mice with PC. The potential mechanisms of action of GLN include maintenance of mucosal integrity, anti-inflammatory effects as a result of increased glutathione synthesis, and induction of heat shock protein synthesis to attenuate cytokines and improve immune competence [12]. 

The previous concern that the use of nutrition support may overstimulate tumor growth was not observed in our study as the mean tumor volume of the pancreatic tumor was smaller in the GLN-treated mice compared to the control group. Moreover, GLN supplementation increased survival time. Within the last 2 decades, several clinical studies have evaluated the tolerance and safety of GLN in cancer patients. In the majority of these clinical studies, orally administered GLN was well tolerated with no evidence of increased tumor growth. It is important that the effect of GLN supplementation on tumor growth receives further validation in both animal and clinical studies. 

The mean serum albumin level was higher in GLN-treated group. This result may support the previous finding that dietary GLN supplementation had a positive influence on visceral protein status [16]. It remains to be elucidated whether increased albumin levels correlate with reduced ascites formation. Since there was no significant difference of serum AST levels between these two groups, the mechanism of albumin increment may not be associated with improvement or protection of liver function. Furthermore, the albumin increment effect of GLN implicates its possible application in management of ascites related to albumin deficiency, such as liver cirrhosis.

Improving the management of ascites in terminal stage pancreatic cancer patients is a critical issue, not only for improvement of end-of-life quality, but also for achieving life-prolonging palliation. This preliminary study demonstrates that oral supplementation of GLN in PC patients may represent a promising immunonutrient for improved ascites control. Further clarification of the mechanism of action as well as clinical validation is warranted.
